# Knowledge and practices of households on safe water chain maintenance in a slum community in Kampala City, Uganda

**DOI:** 10.1186/s12199-019-0799-3

**Published:** 2019-06-14

**Authors:** Charles Ssemugabo, Solomon Tsebeni Wafula, Rawlance Ndejjo, Frederick Oporia, Jimmy Osuret, David Musoke, Abdullah Ali Halage

**Affiliations:** Department of Disease Control and Environmental Health, School of Public Health, Makerere University College of Health Sciences, Kampala, Uganda

**Keywords:** Safe water chain, Maintenance, Households, Slum, Uganda

## Abstract

**Background:**

More than half of the disease burden in Uganda can be prevented through improving water, sanitation, and hygiene (WASH). In slum communities, water supply is insufficient but also highly contaminated; therefore, ensuring that the safe water chain is maintained by households is paramount to preventing water-related diseases. This study aimed at assessing knowledge and practices of households on safe water chain maintenance in slum communities in Kampala City, Uganda.

**Methods:**

This was a community-based cross-sectional study carried out among 395 households in slum communities in Kampala, Uganda. Data were collected using a semi-structured questionnaire. Prevalence ratios (PRs) and their 95% confidence intervals were used as a measure of association between safe water chain management and associated knowledge and practice factors. The PRs were obtained using a multivariable modified Poisson regression with logarithm as the link function, with robust standard errors.

**Results:**

Majority (76.7%, 303/395) of the households collected their water from a piped water system and paid for the water (72.9%, 288/395). Almost all (97.2%, 384/395) of the participants said that they knew the dangers associated with drinking unsafe water, boiled their drinking water (95.4%, 377/395), and used storage containers that minimize contamination (97.0%, 383/395). However, only (32.4%, 128/395) of the households satisfactorily maintained the safe water chain. Female- (adjusted PR = 1.82, 95% CI (1.19–2.78)) and student-led households (adjusted PR = 1.58, 95% CI (1.03–2.41)) and those whose heads had attained post-primary education (adjusted PR = 1.48, 95% CI (1.02–2.17)) were more likely to satisfactorily maintain the safe water chain. This was similar among members who thought most contamination occurs during storage (adjusted PR = 1.47, 95% CI (1.10–1.97)).

**Conclusion:**

Only a third of the households maintained the safe water chain satisfactory. Female-led, student-led, and post-primary educated-led household and household that thought most contamination occurs during storage were more likely to maintain the safe water chain. There is a need to improve the level of awareness about the importance of the safe water chain among slum dwellers.

## Background

Adequate water, sanitation, and hygiene (WASH) is essential to ensure good health and wellbeing. In fact, 85% of the disease burden in Africa could be prevented through improved WASH [1]. Indeed, through improvements in WASH, 502,000, 280,000, and 297,000 deaths due to inadequate drinking water, sanitation, and poor hand hygiene respectively could be averted [2]. Specifically, interventions aimed at improving water quality have been associated with diarrhea and infectious disease reductions [3, 4]. This notwithstanding, many countries including Uganda are still grappling with challenges related to water access with 663 million people in the world estimated to lack access to improved water supplies, half of whom are in sub-Saharan Africa [5]. The situation is worse in slum areas which are usually characterized by inadequate access to water and thus a high burden and episodic outbreaks of WASH-related infections such as typhoid fever, cholera, and dysentery [6–8]. Another key indicator of water supply is water quality, and studies in slum settings found high levels of contamination attributed to inappropriate technology and practices for poor waste disposal [9–11]. Therefore, in addition to ensuring access to safe water particularly in slums, similar efforts should be made to ensure that the provided water is of good quality through maintaining the safe water chain.

Safe water chain includes all processes involved in ensuring that water is not contaminated through all stages from the water source to consumption. Key stages in the safe water chain include water collection, handling, transportation, storage and treatment, and consumption. Although interventions focused on improving household water quality such as improving water storage or treatment have registered positive outcomes in terms of disease reduction [12, 13], measures may not be readily accessible by most households in slums. This further emphasizes the importance of taking practicable measures to avoid water contamination along the water chain. Also, knowledge of communities about safe water chain maintenance interventions and the extent to which they are practiced is important in planning feasible and effective intervention for slum settings. In addition, previous studies in slum settings have shown deficiency in knowledge on WASH among community members [11, 14].

In Kampala, 53.6% of the urban population live in slum settings [15]. Majority of the households in Kampala slums collect their drinking water from the piped water system, regarded as safe, provided by National Water and Sewerage Corporation (NWSC) [16]. However, disease trends in Kampala slums portray them as prone to diarrheal disease outbreaks [7] including a 2014 reported outbreak of typhoid attributed to consumption of contaminated water [17]. These diarrheal disease outbreak occurrences are influenced by household safe water chain practices. This study therefore assessed knowledge and practices of households on safe water chain maintenance in slum communities in Kampala City, Uganda.

## Methods

### Study design and setting

This was a community-based cross-sectional study that used an interviewer-administered semi-structured questionnaire to collect data from household heads or other adults regarding maintenance of the safe water chain. The study was carried out in Kasubi slum, one of the many slums in the outskirts of Kampala, Uganda’s capital city [18]. A slum is defined as a heavily populated urban informal settlement where the inhabitants are characterized by substandard housing and low standard of living [19]. Kasubi parish comprises of mainly informal and substandard housing with a few businesses. It has an estimated population of 384,386 people translating to about 11,372 people/km^2^ spread across its 9 zones [20]. The major sources of water for residents in Kasubi are water taps (stand pipes) with a fee attached for water collection and springs. We purposively selected Kasubi parish due to high population density, uneven terrain, and poor sanitation and hygiene conditions in addition to its close proximity to the central business center of Kampala, hence likely to experience challenges in observing the safe water chain.

### Sample size and sampling procedure

Using the formulae for cross-sectional studies [21], and assuming an alpha of 0.05, power (1-beta) of 0.80, a sampling error of 5%, a non-response rate of 5%, and a statistically conservative prevalence of 50% for households that do not maintain the safe water chain, a final sample size of 401 households was obtained. The 50% prevalence of households which did not maintain the safe water chain was used to obtain an unbiased sample because previous studies carried out in this area were not focused on maintenance of the safe water chain [22–24]. This sample size was distributed proportionately across the six selected zones out of the nine in Kasubi parish based on population size. The number of households in each zone was obtained from Lubaga division offices, and sampling proportionate to size was used to obtain the number of target households from each zone (Table 1). Households, defined by the Uganda Bureau of Statistics (UBOS) as a group of persons who normally live and eat together [25], were selected using systematic random sampling. The number of households in each zone was divided by the number of households to be selected from each zone to create a sampling interval. Within each zone, the first household was selected randomly. Subsequent households were selected by skipping a number of households equivalent to the sampling interval calculated based on the population of the selected zone (Table 1) until the sampled number of households in that zone was achieved.

**Table 1:** Sample size distribution across the zones

Zone	Total number of households	Sampled households per zone
Kawaala 1	3500	100
Kasubi zone 1	2000	64
Kasubi zone 3	2800	84
Kawaala 2	2400	67
Kasubi zone 4	1700	50
Kasubi zone 2	1600	36

### Data collection

Data were collected using an interviewer-administered semi-structured questionnaire. We asked respondents about their sources of domestic water, knowledge on safe water chain, and maintenance of safe water chain. The questionnaire was developed based on reviewed literature on safe water chain [26–31]. Data collection tools were pretested in Mulago slum within the city which had similar characteristics with the study area. Trained research assistants who were Environmental Health Students of Makerere University collected the data from all selected households.

### Data management and analysis

Data were examined and cleaned daily during collection for completeness and entered in EpiData version 3.02 (EpiData association; Denmark). We used Stata 13.0 (Statacorp Texas; USA) for analysis. To determine the status of safe water chain maintenance (outcome variable), which was classified as either high maintenance or low maintenance, nine questions were asked on practices on safe water chain maintenance with responses “Yes” assigned 1 and “No” assigned 0 during analysis. Respondents who had a total score of at least 7 of the 9 were considered to have high maintenance of safe water chain practices and the rest otherwise. Prevalence ratios (PRs) computed using a generalized linear model of the Poisson family with the logarithm as the canonical link function, with robust standard errors while applying a forward elimination method, were used to measure the association between the outcome and independent variables. PRs were used instead of odds ratios since the prevalence of the outcome variable was > 10%, yet logistic regression’s odds ratios tend to overestimate the relative risk in such instances [32, 33]. Simple models consisting of the outcome and one independent variable were run to obtain the crude PRs. In the multivariable model, variables that had *p* values of up to 0.1 were included. The crude and the adjusted PRs and their corresponding 95% confidence intervals are presented.

### Ethical considerations

Ethical approval for the study was obtained from the Makerere University School of Public Health Higher Degrees, Research and Ethics Committee (101). The study was also approved by Uganda National Council of Science and Technology registration (HS 867). Participation in the study was voluntary, and household heads or other consenting adults provided written informed consent.

## Results

### Sociodemographic characteristics of participants

A total of 395 households participated in the study out of the 401, resulting in a response rate of 98.5%. Majority of the participants were females (75.9%, 300/395) and Christians (77.5%, 306/395), had attained post-primary education (69.1%, 273/395), and aged 18–29 years (63.3%, 250/395). Most (38.5%, 152/395) household heads were engaged in business (Table 2).

**Table 2:** Sociodemographic characteristics of participants

Variables	Frequency (*n* = 395)	Percentage
Gender		
Female	300	75.9
Male	95	24.1
Age (years) [mean (± SD)]	30.0 (10.8)	
18–29	250	63.3
30–45	104	26.3
≥ 46	41	10.4
Education level		
None or primary	122	30.9
Post-primary	273	69.1
Marital status		
Single	148	37.5
Married	207	52.4
Widowed/separated/divorced	40	10.13
Religion		
Christian	306	77.5
Muslim	89	22.5
Occupation		
Business	152	38.5
Unemployed	103	26.1
Formal employment	49	12.4
Student/pupil	45	11.4
Farming	46	11.7
Household size		
1–3	174	44.0
4–6	162	41.0
≥ 7	59	15.0

### Sources and institutional aspects of domestic water

Majority (76.7%, 303/395) of the households used piped water as their source of water for domestic purposes, whereas only (23.3%, 92/395) obtained water from springs. All households were located within 500 m to the nearest water source, with (70.4%, 278/395) of the participants moving a distance of less 20 m to collect water. Only (25.1%, 99/395) of the households obtained water from communally owned sources. Most (61.5%, 243/395) households had daily water per capital utilization of less than 40 l and a large proportion (72.9%, 288/395) of participants paid money to collect water. Among the communal water sources, more than half (53.5%, 53/99) had water user committees (Table 3).

**Table 3:** Sources of domestic water and their maintenance

Variables	Frequency (*N* = 395)	Percentage
Main water source		
Piped water	303	76.7
Springs	92	23.3
Estimated distance to nearest water source (meters)		
≤ 20 (within the compound)	278	70.4
21–100	85	21.5
101–500	32	8.1
Water obtained from communally owned source		
No	296	74.9
Yes	99	25.1
Water user committee for communal sources present (*n* = 99)		
Yes	53	53.5
No	27	27.3
Do not know	19	19.2
Water consumption per person per day (liters)		
≤ 40	243	61.5
> 40	152	38.5
Paid for water		
Yes	288	72.9
No	107	27.1
Paid towards maintenance of main water source		
No	368	93.2
Yes	27	6.8

### Knowledge on water safety and its importance

Half (50.1%, 198/395) of the study participants said they knew that most contamination of water occurred at the water source. When asked whether they knew the dangers associated with drinking unsafe water, majority (97.2%, 384/395) of the participants said they did and (61.8%, 244/395) indicated that boiling drinking water was key to preventing diarrheal diseases (Table 4).

**Table 4:** Knowledge on water safety and its importance

Variables	Frequency (*N* = 395)	Percentage
Contamination of water occurs		
At the source	198	50.1
During storage (storage container)	117	29.6
During use	49	12.4
Do not know	31	7.9
Safe water is		
Water that is clear	221	56.0
Boiled water	89	22.5
Water that has no germs	55	13.9
Did not know	30	7.6
Said they knew dangers of drinking unsafe water		
Yes	384	97.2
No	11	2.8
Benefits of drinking safe water		
Prevents disease	370	94.4
Others (saving money, improving work efficiency)	10	2.5
Did not know	15	3.8
Preventive measures for diarrheal diseases		
Drinking boiled water	244	61.8
Keeping good personal hygiene	38	9.6
Eating well-cooked food	26	6.6
Washing hands with soap before eating food	23	5.8
Others*	30	7.6
Did not know	34	8.6

*Other preventive measures included bathing regularly, washing food, and proper waste disposal

### Maintenance of safe water chain by households

Majority of the households used appropriate water collection containers such as jerry cans or pots (97.0%, 383/395). Majority of these water collection containers were clean (81.0%, 320/395). In addition, almost all (95.4%, 377/392) participants said they boiled their water to make it safe for drinking. Most households used storage containers which were covered (88.6%, 350/395) and clean (95.4%, 377/395). However, only (32.4%, 128/395) maintained the proper safe water chain practices (Table 5).

**Table 5:** Practices on safe water chain maintenance

Variables	Frequency (*n* = 395)	Percentage
Used water collection container that minimizes contamination^1^		
Yes	383	97.0
No	12	3.0
Water collection container clean^1^		
Yes	320	81.0
No	75	19.0
Methods of drinking water treatment^^^		
Boiling	377	95.4
Chlorination	16	4.1
Filtration	09	2.3
Do not treat water	11	2.8
Method of water treatment appropriate^1^		
No	13	3.3
Yes	382	96.2
No	45	11.4
Yes	350	88.6
Water storage container clean^1^		
No	18	4.6
Yes	377	95.4
Cleaned drinking water storage containers at least once a week^1^		
Yes	108	27.3
No	287	72.7
Cleaned water storage containers by scrubbing and rinsing^1^		
No	174	44.0
Yes	221	56.0
Used a separate cup or container to draw drinking water from storage containers^1^		
No	329	83.3
Yes	66	16.7
Maintenance of safe water chain (mean score, SD)	6.91 ± 1.28	
Low (scores < 6.91)	267	67.6
High (scores ≥ 6.91)	128	32.4

^1^Variables used in determining average safe water chain practice scores

^^^Multiple options

### Factors associated with maintenance of the safe water chain

The proportion of households with high maintenance of safe water chain practices was higher among female-led households (adjusted PR = 1.82, 95% CI (1.19–2.78)) and those whose heads had attained post-primary education (adjusted PR = 1.48, 95% CI (1.02–2.17)) and those that were student-led (adjusted PR = 1.58, 95% CI (1.03–2.41)) when compared with their counterparts. Households whose heads thought that most contamination occurred during storage were 50% (adjusted PR = 1.47, 95% CI (1.10–1.97)) more likely to maintain safe water chain practices compared to those who thought it occurred at the water source (Table 6).

**Table 6:** Factors associated with maintenance of the safe water chain

Characteristic (categories)	Safe water chain maintenance		Crude PR (95% CI)	*p* value	Adjusted PR (95% CI)	*p* value
	Yes, *n* (%)	No, *n* (%)				
Socio-economic factors						
Gender						
Male	20 (21.1)	75 (78.9)	1		1	
Female	108 (36.0)	192 (64.0)	1.71 (1.13–2.56)	0.012*	1.82 (1.19–2.78)	0.005*
Age (years)						
14–29	90 (36.0)	160 (64.0)	1		1	
30–45	27 (26.0)	77 (74.0)	0.72 (0.50–1.04)	0.079	0.84 (0.59–1.22)	0.377
> 45	11 (26.8)	30 (73.2)	0.75 (0.44–1.27)	0.279	1.06 (0.62–1.81)	0.842
Education level						
None/primary	27 (22.1)	95 (77.9)	1		1	
Post-primary	101 (37.0)	172 (63.0)	1.67 (1.16–2.41)	0.006*	1.48 (1.02–2.17)	0.041*
Marital status						
Single	51 (34.5)	97 (65.5)	1			
Married	68 (32.9)	139 (67.2)	0.95 (0.71–1.28)	0.751		
Widowed/divorced/separated	9 (22.5)	31 (77.5)	0.65 (0.35–1.21)	0.176		
Occupation						
Business	40 (26.3)	112 (73.7)	1		1	
Unemployed	33 (32.0)	70 (68.0)	1.21 (0.83–1.79)	0.320	1.13 (0.78–1.64)	0.528
Salaried work	20 (40.8)	29 (59.2)	1.55 (1.01–2.38)	0.045*	1.41 (0.94–2.12)	0.098
Student	21 (46.7)	24 (53.3)	1.77 (1.18–2.67)	0.006*	1.58 (1.03–2.41)	0.034*
Farming	14 (30.4)	32 (69.6)	1.16 (0.69–1.93)	0.578	1.22 (0.73–2.05)	0.453
Number of people in the household						
1–3	52 (29.9)	122 (70.1)	1			
4–6	54 (33.3)	108 (66.7)	1.12 (0.81–1.52)	0.497		
≥ 7	22 (37.3)	37 (62.7)	1.24 (0.83–1.86)	0.281		
Water source-related and individual factors						
Main water source used by household						
Piped water	99 (32.7)	204 (67.3)	1			
Springs	29 (31.5)	63 (68.5)	0.96 (0.69–1.36)	0.837		
Water from communally owned source						
No	102 (34.5)	194 (65.5)	1			
Yes	26 (26.3)	73 (73.7)	0.76 (0.53–1.10)	0.146		
Estimated distance to water source (meters)						
≤ 20 (within the compound)	94 (33.8)	184 (66.2)	1			
21–100	23 (27.1)	62 (72.9)	0.80 (0.54–1.18)	0.258		
101–500	11 (34.4)	21 (65.6)	1.02 (0.61–1.68)	0.949		
Water consumption per person per day (liters)						
≤ 40	72 (29.6)	171 (70.4)	1			
> 40	56 (36.8)	96 (63.2)	1.24 (0.94–1.65)	0.134		
Paid for water collection/fetching						
No	41 (38.3)	66 (61.7)	1			
Yes	87 (30.2)	201 (69.3)	0.79 (0.59–1.06)	0.118		
Perception of where most contamination occurred						
At the source	58 (29.3)	140 (70.7)	1		1	
During storage (jerry can/container)	53 (45.3)	64 (54.7)	1.55 (1.15–2.08)	0.004*	1.47 (1.10–1.97)	0.009*
At point of use	10 (20.4)	39 (79.6)	0.70 (0.38–1.26)	0.233	0.69 (0.39–1.22)	0.197
Did not know	7 (22.6)	24 (77.4)	0.77 (0.39–1.53)	0.458	0.79 (0.41–1.52)	0.475

Level of confidence = 95%; gender, age, education, and occupation were the potential confounders for safe water chain maintenance

*PR* prevalence ratio, *CI* confidence interval

*p < 0.05

## Discussion

This study assessed knowledge and practices of households on safe water chain maintenance in households in Kasubi slum in Kampala, Uganda. Our findings show that the major sources of domestic water were private tap stands and protected springs. Most of the households paid for water, treated their water for drinking by boiling, and knew the sources of water contamination and dangers of drinking contaminated water. However, only a third of the households reported practices that maintain the safe water chain. Household heads who were females and students and/or attained post-primary education were more likely to maintain the safe water chain. Household heads who said that most contamination of water happens during storage were also more likely to maintain the safe water chain.

The major sources of domestic water in our study were private taps (71.6%) and protected springs (20.5%). The use of private tap stands is not surprising because many areas within the city including slums in Kampala are supplied with piped water from NWSC, a government agency responsible for treatment and distribution of water to the public. Similar studies done in Ghana and India have also found tap stands as a popular water source in slums [34, 35]. Slums most likely occur in low lying areas were protected springs are usually located. Protected springs and tap water are generally considered improved water sources and are therefore expected to provide relatively good quality water [36]. However, recent studies in Kampala showed that most protected springs were contaminated [10, 37]. Most households paid water bills which is expected in an urban setting since majority are connected to piped water which are metered and paid for by the final consumer.

From our study, most of the household heads knew the different ways by which water could get contaminated. Majority of respondents also knew the dangers of drinking contaminated water such as increased risk of diarrheal diseases. Since slums in Kampala have in the past experienced frequent outbreaks of diarrheal diseases especially cholera and typhoid [38], the high level of knowledge could be attributed to the intense awareness campaigns that are conducted whenever these outbreaks occur. In our study, only one third of the households maintained safe water chain management practices. This implies that two thirds of the population in slums in Kampala are at risk of drinking unsafe water and acquiring diarrheal diseases due to lack of maintenance of the safe water chain. Our findings corroborate with findings from a study in India where majority of the urban population did not observe safe water chain practices [39]. Therefore, there is a need to increase slum communities’ awareness on maintenance of the safe water chain.

Most households used collection and storage containers that would minimize contamination which is a good practice. However, few households were cleaning their containers regularly. In addition, most of the containers were not covered, and only a few households used a separate cup to draw drinking water from the containers (16.7%). This practice is sometimes discouraged with preference for small-mouthed containers. Educating people about the risk and pathways of water contamination can help improve water quality and consequently mitigate risks of diarrheal diseases. Another finding from our study is that boiling was the most common method of treatment of drinking water. This finding is similar to that from another Ugandan study that established that majority (89%) of the households were boiling their drinking water [40]. Boiling is known to be the most popular water treatment method especially in low-income countries [41]. It is a reliable treatment method against microbial agents if well used, and water thereafter well stored [41, 42]. Practicing such simple and cheap interventions at household level can lead to an improvement in the quality of drinking water which eventually leads to reduction in diarrheal diseases [43].

Female-headed households were more likely maintain safe water chain management practices. Our finding is in line with a study conducted in Cameroon which indicated that female-headed households were more likely to invest in the effort of fetching clean water and ensuring proper storage [43]. It has also been shown that women often engage in water collection, storage and treatment, and use compared to men in communities especially in slums [44, 45]. Women are at higher risk of water-borne and water-based infections such as diarrhea, ascariasis, and trichuriasis than men, as such observing high standards of safe water chain to minimize water-borne disease risk is imperative to them [46, 47]. Student-led households were more likely to observe the safe water chain as compared to those who were engaged with business. This finding was understandable as students are likely to be routinely taught WASH aspects at school. Indeed, some studies have demonstrated that students can learn many things at school and influence behavior change in their homes and communities [48, 49]. This shows that education can influence household practices on safe water chain. Household heads who had attained post-primary education were more likely to observe safe water chain compared to those with primary or no education. This finding is in line with studies [50–52] which indicated that high levels of education result in adoption of better decisions on safe water management. Educational awareness programs on safe water chain are needed to benefit individuals with low education status and consequently minimize risk due to poor safe water practices.

Contamination of water can occur at any point in the water chain from the source to the point of use [53, 54]. In our study, household heads who thought most contamination occurs during storage were more likely to observe safe water chain compared to those who thought it occurs more at the source. It is known that significant recontamination of water can occur through drawing it with cups and hands as reported in other studies [55, 56]. Evidence also shows that point-of-source bacterial contamination may be rare when water is obtained from standpipes or taps as in the case of this setting. In fact, many city water supplies are treated centrally in conventional systems but contamination could occur mostly through unsafe water storage [57, 58]. However, there is a need to educate slum dwellers on critical safe water chain practices that need to be maintained along the entire drinking water chain as demonstrated in an Ethiopian study on the effect of WASH on childhood illnesses [59].

Our study is limited by the fact that all practices reported about were self-reported and could have been subject to social desirability bias. However, the study makes a significant contribution regarding safe water chain maintenance in an urban slum which has rarely been researched. The study findings could also be generalizable to other slums in Kampala city as these have been reported to be similar in context.

## Conclusion

Knowledge on the safe water chain was generally satisfactory although only a third of the households maintained the safe water chain. Female-headed households, post-primary educated household heads, and student-led households were significantly more likely to maintain the safe water chain. Therefore, there is a need to improve safe water chain practices among slum household through continuous health education on the importance of using and drinking safe water.

## Data Availability

The dataset used during the study is available from the corresponding author on reasonable request.

## Abbreviations

NWSC: National Water and Sewerage Cooperation
PRs: Prevalence ratios
UBOS: Uganda Bureau of Statistics
WASH: Water, sanitation, and hygiene
